# Plerixafor and G‐CSF Versus Placebo and G‐CSF in Moderately Mobilizing Patients With Multiple Myeloma to Optimize Hematopoietic Stem Cell Collection Results (OPTIMIZE)

**DOI:** 10.1002/jca.70188

**Published:** 2026-10-12

**Authors:** Worel Nina, Schreder Martin, Tanzmann Andreas, Kurz Martin, Huber‐Kraßnitzer Bianca, Neumeister Peter, Rosskopf Konrad, Sareban Nazanin, Branka Manuela, Kolovratova Vera, Oesterreicher Sophia, Koerbler Beatrix, Kitzweger Elvira, Ruckser Reinhard, Rabitsch Werner, Agis Hermine, Krauth Maria, Greinix Hildegard T.

**Affiliations:** ^1^ Department of Transfusion Medicine and Cell Therapy Medical University Vienna Vienna Austria; ^2^ Department of Internal Medicine I Center for Oncology and Hematology, Clinic Ottakring Vienna Austria; ^3^ Division of Hematology Medical University of Graz Graz Austria; ^4^ Department of Blood Group Serology and Transfusion Medicine Medical University of Graz Graz Austria; ^5^ Department of Internal Medicine II Clinic Donaustadt Vienna Austria; ^6^ Department of Internal Medicine 1, BMT‐Unit Medical University of Vienna Vienna Austria; ^7^ Department of Internal Medicine I, Division of Hematology & Hemostaseology Medical University of Vienna Vienna Austria

**Keywords:** autologous stem cell transplantation, colony‐forming units, hematopoietic stem cell mobilization, multiple myeloma, plerixafor, stem cell graft composition

## Abstract

Efficient collection of hematopoietic progenitor cells (HPCs) is essential for autologous stem cell transplantation (ASCT) in multiple myeloma (MM). Although plerixafor is routinely used in poor mobilizers, its role in patients with intermediate mobilization capacity remains unclear. In this randomized, double‐blind, placebo‐controlled phase IV trial, MM patients with peripheral blood (PB) CD34^+^ counts of 15–40 cells/μL after steady‐state G‐CSF mobilization were randomized to receive plerixafor or placebo. The primary endpoint was successful collection of ≥ 6.0 × 10^6^ CD34^+^ cells/kg, sufficient for two ASCTs, during a single apheresis session. Secondary endpoints included CD34^+^ kinetics, graft composition, colony‐forming capacity, engraftment, immune reconstitution, and safety. Twenty‐one patients were enrolled before premature study termination due to changes in first‐line MM therapy and the COVID‐19 pandemic. Plerixafor significantly increased PB CD34^+^ cell counts compared with placebo (*p* < 0.001). All patients receiving plerixafor achieved the primary endpoint, whereas none in the placebo group achieved it (*p* < 0.001). Mean CD34^+^ yield was significantly higher with plerixafor (10.4 ± 1.9 × 10^6^/kg vs. 2.9 ± 1.3 × 10^6^/kg; *p* < 0.001). Plerixafor‐mobilized grafts contained significantly higher numbers of primitive hematopoietic progenitor subsets and demonstrated increased CFU‐GM formation, indicating enhanced clonogenic potential. Despite marked differences in graft quantity and quality, engraftment kinetics, graft sustainability, and immune reconstitution were comparable between groups. In MM patients with intermediate mobilization capacity, the addition of plerixafor markedly improved stem cell mobilization efficiency and enriched grafts with functionally robust progenitor cells. These findings suggest that selected patients beyond conventional poor mobilizers may benefit from plerixafor‐assisted mobilization.

**Trial Registration:** EudraCT nr.: 2016‐004122‐41

## 1 Introduction

Multiple myeloma (MM) is a hematologic malignancy characterized by the clonal expansion of plasma cells in the bone marrow. Despite recent therapeutic advancements including proteasome inhibitors, immunomodulatory drugs, and monoclonal antibodies (moAb), MM remains largely incurable [1, 2]. For transplant‐eligible patients, autologous hematopoietic stem cell transplantation (ASCT) plays a crucial role in achieving deeper and more durable remissions. Although challenged by recent trial results, tandem transplantation involving two sequential ASCTs has often been employed for high‐risk patients or those with suboptimal responses to initial therapies, offering improved progression‐free and overall survival [3, 4, 5].

The success of tandem ASCT depends on the efficient collection of an adequate number of hematopoietic progenitor cells (HPCs), ideally obtaining sufficient CD34+ cells for both transplants in a single mobilization and apheresis session. Mobilization failure or suboptimal HPC yield, commonly defined as < 2.0 × 10^6^ CD34+ cells/kg for a single ASCT or < 4.0 × 10^6^ CD34+ cells/kg for tandem ASCT, remains a clinical challenge [6]. So far, a universally accepted threshold of HPC in the peripheral blood (PB) to distinguish between good and poor mobilizers has not been clearly defined; however, a PB CD34+ cell count of < 20 cells/μL is often used in clinical practice to define poor mobilization, complicating clinical decision‐making. Mohty et al. describes three categories based on CD34 + levels in PB before leukapheresis: ‘borderline’ poor mobilisers (11–19/μL at maximum stimulation), ‘relative’ poor mobilisers (6–10/μL) and ‘absolute’ poor mobilisers (< 5/μL) [7].

Granulocyte‐colony stimulating factor (G‐CSF) either as a monotherapy or in combination with chemotherapeutic agents remains the cornerstone of HPC mobilization. However, a significant proportion of patients with MM exhibit suboptimal mobilization, defined as the inability to collect a sufficient number of CD34+ cells for transplantation. These suboptimal mobilizers are particularly those with prior exposure to lenalidomide or alkylating agents [6, 7]. In September 2019, daratumumab, a moAb targeting CD38, was approved for newly diagnosed MM patients based on two landmark phase 3 clinical studies evaluating the addition of daratumumab to standard‐of‐care front‐line treatments for newly diagnosed, transplant‐eligible multiple myeloma—CASSIOPEIA and PERSEUS trial—and since then has been increasingly used in induction regimens for MM patients undergoing ASCT [2, 8, 9]. While this strategy has significantly improved treatment outcomes, these studies have reported an unfavorable impact of daratumumab‐containing induction therapy on stem cell mobilization, including reduced CD34^+^ cell yields, increased plerixafor requirements, and prolonged apheresis procedures [10, 11].

The integration of plerixafor, a CXCR4 antagonist, into mobilization regimens has emerged as a potent adjunct to G‐CSF and significantly improved mobilization outcomes by disrupting the stromal‐derived factor‐1 (SDF‐1)/CXCR4 axis and releasing HPCs into the circulation [12, 13]. Clinical studies have demonstrated that combining plerixafor with G‐CSF not only enhances mobilization success rates but also reduces the need for additional apheresis sessions and mobilization approaches. Plerixafor has been approved by the European Medicines Agency (EMA) specifically for use in patients with MM and malignant lymphoma, whose cells mobilize poorly [14]. Therefore, plerixafor is commonly used in European countries, either in a remobilization setting after unsuccessful prior mobilization or pre‐emptively to support an ongoing mobilization strategy in patients at risk of mobilization failure. Routine use of plerixafor for all MM patients, as licensed by the Food and Drug Administration (FDA) in the US, is not common practice in Europe and is restricted by its high costs, especially in patients who do not meet criteria for poor mobilization and may nevertheless achieve adequate stem cell yields, even if multiple aphereses are required [7, 14, 15, 16].

This randomized, double‐blind, placebo‐controlled trial enrolled MM patients with predefined intermediate mobilization potential (defined as a PB CD34+ count of ≥ 15 and ≤ 40 cells/μL) and evaluated the efficacy of steady‐state G‐CSF plus plerixafor versus steady‐state G‐CSF plus placebo for achieving collection of ≥ 6.0 × 10^6^ CD34+ cells/kg, sufficient for two ASCTs, within a single apheresis session. By examining mobilization efficiency, safety, and cost‐effectiveness, this study aims to refine mobilization protocols to optimize resource use while ensuring clinical efficacy.

## 2 Materials and Methods

### 2.1 Study Design and Patients

We conducted a phase IV, multicenter, randomized, double‐blind, placebo‐controlled study in MM patients with steady‐state HPC mobilization.

Primary study endpoint was the proportion of patients achieving ≥ 6.0 × 10^6^ CD34+ cells/kg in one apheresis session for a planned double ASCT. CD34+ cell yields were determined in local laboratories and used to calculate efficacy endpoints. Secondary endpoints were (i) fold‐increase in PB CD34+ cells immediately before apheresis after application of placebo or study drug; (ii) cell composition of leukapheresis products including distribution of myeloid and lymphoid subpopulations, and NK‐cells [17, 18], (iii) collection efficiency (CE1 and CE2); (iv) comparison of colony forming units CFU‐GM; (v) the number of days to neutrophil and platelet engraftment; and (vi) proportion of patients with sustainable graft and immune reconstitution assessed at 15 and 28 days, 3 months, and 6 months after ASCT. Side‐effects of placebo/study drug were documented.

The study was conducted in accordance with the Declaration of Helsinki and Good Clinical Practice guidelines and approved by the competent authorities and ethics committee of each participating institution (EK 2274/2016; EudraCT Nr.: 2016–004122‐41). Sanofi Genzyme provided financial support for study conduct but had no role in data analysis, interpretation, or manuscript preparation.

Patients with an age of 18 to ≤ 75 years with biopsy‐confirmed diagnosis of MM in whom HPCs for at least two ASCTs had to be collected after steady‐state mobilization with G‐CSF alone and with ≥ 15/μL and ≤ 40/μL CD34+ cells detected in the PB on day 4 of G‐CSF administration were eligible to participate. Additional inclusion criteria were: at least 4 weeks since end of the last cycle of chemotherapy, absolute neutrophil count (ANC) of ≥ 0.75 × 10^9^/L, platelet count more than 50 × 10^9^/L, cardiac and pulmonary status sufficient to undergo leukapheresis and transplantation, HIV negativity, and in agreement to use an approved form of contraception if of childbearing potential.

Patients were excluded if they had hypersensitivity to the active study drug; a history of clinically significant cardiac abnormality or arrhythmia; liver function test abnormalities > 3× upper limit of normal for aspartate amino transferase (AST), alanine amino transferase (ALT), total bilirubin; any active infection including hepatitis B or C; fever > 38.5°C; history or signs of acute and chronic leukemia or myelodysplastic syndrome. In addition, patients were excluded if they received chemomobilization or were pregnant or breastfeeding. Patients with moderate and severe renal insufficiency (CrCl ≤ 50 mL/min) had the plerixafor dose reduced to 0.16 mg/kg.

Patients were informed about the study before start of steady‐state mobilization and that they may not be eligible for the study if their day 4 CD34+ cell count in the PB is < 15/μL or > 40/μL. All participating patients provided written informed consent and could withdraw from the study at any time.

### 2.2 Treatment Plan and Apheresis

Patients were randomized 1:1 to receive either plerixafor 0.24 mg/kg or placebo on day 4 of G‐CSF if they fulfilled the study requirements. The mobilization regimen used was a steady‐state mobilization with G‐CSF alone at a dose of 2 × 5 μg/kg per day starting on day 1. On day 4 the morning G‐CSF injection was given at 06:00 am and at least 2 h thereafter PB CD34+ cell count was analyzed. Plerixafor or placebo was given either between 10:00 and 12:00 with leukapheresis starting 4–6 h thereafter on day 4 of G‐CSF or between 17:00 to 22:00 with leukapheresis starting 8–10 h thereafter on day 5 in the morning depending on the availability of apheresis slots. In case of evening application of plerixafor or placebo, G‐CSF was continued until Day 4 in the evening (Figure S1).

CD34+ collection was performed with a Spectra Optia apheresis device (Terumo BCT), Software Version 11, processing 3 times patients' total blood volume. For anticoagulation ACD‐A was used at a ratio of 1:12. Prophylactic calcium supplementation was given according to institutional standards. The apheresis product was further cryopreserved and stored following local practice guidelines at each study center.

If the primary endpoint of ≥ 6 × 10^6^/kg CD34+ cells was not reached in one collection, a second and if needed third collection procedure was performed on consecutive days. However, only the first leukapheresis procedure was included in the analyses.

### 2.3 Flow Cytometry Analyses and Colony Forming Unit Assays

Flow cytometry analyses were performed as described elsewhere [19]. We aimed to compare differences in the CD34+ subgroups between the plerixafor and placebo group. Therefore, we analyzed multipotent progenitors (MPPs; the earliest CD34+ subset), lymphoid‐primed multipotent (LMPP; harbor the potential to create neutrophil and macrophage progenitors (GMPs), multi‐lymphoid precursors (MLPs)) and erythromyeloid progenitors (EMP) [17, 19]. Analyses were performed on a FACS Fortessa (BD Biosciences) equipped with four solid‐state lasers with excitation wavelengths (nm) of 488, 405, 561, and 640. Computer software (FACSDiVa 6, BD Biosciences) was used for data acquisition and evaluation. For each analysis 150 000 CD45+ events were acquired (Supporting Information).

In vitro stem cell assays were performed on a thawed reference vial within 72 h of cryopreservation. A commercially available kit (MethoCultTM GF H84434, Stemcell Technologies, Vancouver, Canada) was used according to the manufacturer's instructions [20]. In brief, cryopreserved vials were thawed, diluted and 2 × 10^4^ viable nucleated cells (NC) were plated together with cytokines under aseptic conditions. The plates were incubated for 14 days and colony forming unit‐granulocyte‐macrophage (CFU‐GM), burst forming unit‐erythroid (BFU‐E), and CFU‐granulocyte erythrocyte macrophage megakaryocyte (CFU‐GEMM) colonies were analyzed with an inverted microscope (Supporting Information).

### 2.4 Autologous Stem Cell Transplantation

Patients underwent conditioning regimen consisting of either melphalan 70 or 100 mg/m^2^/day on 2 consecutive days (day −4 and −3) in preparation for ASCT according to local standard of care. A minimum of 2 × 10^6^ CD34+ cells/kg was required for a single transplantation. Engraftment was defined as the first of 3 days with ANC above 0.5 × 10^9^/L, leukocytes > 1.0 × 10^9^/L, and platelets > 20 × 10^9^/L without a transfusion for the preceding 7 days. Graft sustainability was defined as maintenance of neutrophil counts of more than 1.0 × 10^9^/L with no G‐CSF support for at least 1 week, platelet count of more than 50 × 10^9^/L, and hemoglobin level of ≥ 10 g/dL during the first 6 months. Graft sustainability and immunologic recovery were analyzed at day 15, 28, month 3, and month 6 (Table S1). To augment engraftment, patients received G‐CSF at a dose of 5 μg/kg/day according to local guidelines.

If a tandem transplantation was performed within 6 months of the first transplantation, engraftment data from the second transplantation were used to compare outcomes between the 2 groups. For each individual patient, the participation in the study ended 6 months after the first or second ASCT, respectively.

### 2.5 Safety

All patients who received one dose of study medication (placebo or study drug) were included in the safety analyses. The severity of toxicities was graded according to the active version of National Cancer Institute (NCI) Common Terminology Criteria for Adverse Events (CTCAE, Version 5.0). Safety of the mobilizing regimen was monitored by the incidence of adverse events, serious adverse events, and changes from baseline in medical history, clinical laboratory measurements, vital signs, and findings of physical examination.

### 2.6 Statistical Analysis

Based on published data we estimated that 40% of patients in the placebo group will achieve the primary endpoint [21, 22]. Therefore, a sample size of 42 patients per group was needed to provide a power of 80% to detect a 30% difference between the 2 treatment arms (placebo and plerixafor). The calculations were performed with a significance level *α* 0.05. To balance an assumed dropout rate of 10% an inclusion of a total of 90 patients (45 patients in the placebo arm and 45 patients in the treatment arm) was planned.

The intent‐to‐treat analysis, which includes all randomized patients, was used to establish mobilization efficacy. The Chi‐Square‐test was used to compare the number of aphereses needed to achieve the primary endpoint. Differences in graft composition of stem cell products, engraftment time, graft sustainability and immunologic recovery were assessed with the unpaired *t*‐test. If the cell counts were not normally distributed according to the Shapiro–Wilk‐Test, the Man‐Whitney‐U‐Test was performed. A two‐tailed *p* value of 0.05 is considered statistically significant. All analyses were carried out using statistical software SPSS (version 28.0, SPSS Inc., Chicago, IL, USA).

## 3 Results

Unfortunately, only 21 patients (10 assigned to plerixafor and 11 to placebo) were enrolled at two study sites between February 2018 and 2021. The main factors limiting recruitment were the approval of daratumumab for standard first‐line therapy of newly diagnosed MM patients, which has been associated with impaired stem cell mobilization outcomes and the COVID‐19 pandemic [10, 11]. Daratumumab‐containing induction regimens have been associated with reduced peripheral blood CD34+ cell yields, an increased need for plerixafor rescue, a higher number of apheresis sessions, and lower overall stem cell collection efficiency compared with daratumumab‐free regimens. Consequently, a larger proportion of patients either failed to meet the study's predefined mobilization criteria or required plerixafor as part of standard clinical care, thereby substantially reducing the pool of eligible participants [10, 11]. Therefore, after an interim analysis of the first 21 patients, it was decided to stop further enrollment.

We report herein the data of all included patients through 6 months of follow‐up. Overall, patient demographics and baseline characteristics were similar between the 2 groups (Table 1).

**TABLE 1:** Patient demographics and baseline characteristics.

	Plerixafor *N* = 10	Placebo *N* = 11	*p*
Age, years (median, range)	57 (45–68)	59 (51–68)	n.s.
Male, *n* (%)	7 (70)	7 (63)	n.s.
Time from diagnosis to study enrollment, months (median, range)	5 (2–16)	6.6 (3–23)	n.s.
International staging system (ISS)			n.s.
I	5	5	
II	3	6	
III	2	0	
Paraprotein, *n* (%)			n.s.
IgG kappa/lambda *n* (%)	3 (30)/1 (10)	4 (36)/3 (27.5)	
IgA kappa/lambda *n* (%)	0/2 (20)	1 (9)/0	
Lambda light chain *n* (%)	4 (40)	3 (27.5)	
Pretreatment			n.s.
VRd, VCd *n*	9	7	
D‐VCD *n*	1	1	
KRd, KCd *n*	0	3	
Prior radiotherapy			n.s.
Yes	3	6	
No	7	5	

Abbreviations: D = daratumumab; KCd = carfilzomib, cyclophosphamide, dexamethasone; KRd = carfilzomib, lenalidomide, dexamethasone; *n* = number; VCd = bortezomib, cyclophosphamide, dexamethasone; VRd = bortezomib, lenalidomide, dexamethasone.

### 3.1 Mobilization and Collection Results

Peripheral blood CD34+ cell counts before study treatments (day 4) was similar between groups (median and range, 29.7 CD34+/μL, 16.9–34.8/μL in the plerixafor vs. 22.0 CD34+/μL, 15.3–38.4/μL in the placebo group; *p* = 0.11; Table 2, Figure 1A). Depending on apheresis slot availability, three patients in the plerixafor group and two in the placebo group underwent apheresis on day 4 of G‐CSF, after receiving 7 doses of G‐CSF. The remaining seven patients in the plerixafor and nine in the placebo group underwent apheresis on day 5, after receiving 8 doses of G‐CSF. Four to 15 h after study treatment, plerixafor‐treated patients had significantly higher PB CD34+ cell counts (median 93.3 CD34+/μL, range 53.4–140 CD34+/μL) than placebo‐treated patients (median 29.3 CD34+/μL, range 11.0–56.2 CD34+/μL; *p* < 0.001; Table 2, Figure 1A). The fold increase in PB CD34+ cell count was also significantly higher in the plerixafor group than in the placebo group, with a median of 3.6‐fold (range, 2.2–6.4) versus 1.2‐fold (range, 0.5–2‐8), respectively (*p* < 0.001; Table 2, Figure 1A). On the first apheresis day all patient in the plerixafor group had PB CD34+ cell counts > 50/μL compared with only one patient (9%) in the placebo group, respectively. In the placebo group 9 patients (82%) had > 20 CD34+ cells/L before start of collection.

**TABLE 2:** Apheresis and engraftment data.

Date given in median, range	Plerixafor	Placebo	*p*
Number of apheresis, *n*	1 (1–1)	2 (2–3)	< 0.001
Patient's total blood volume L	5.2 (3.2–6.8)	5.4 (3.8–6.2)	0.266
Blood volume processed, L	15.5 (9.7–20.3)	17.1 (11.3–18.6)	0.277
Peripheral blood CD34+ cells/μL			
Before plerixafor/placebo	29.7 (16.9–34.8)	22.0 (15.3–38.4)	0.106
Before first apheresis	93.3 (53.4–140)	29.3 (11.0–56.2)	< 0.001
X‐fold increase of CD34+ cells/μL in peripheral blood	3.6 (2.2–6.4)	1.2 (0.5–2.8)	< 0.001
CD34+ cells × 10^6^/kg in the 1st leukapheresis	10.3 (7.8–13.2)	2.52 (1.4–5.3)	< 0.001
Collection efficiency (CE)			
CE2 (%)	58.8 (40.3–93.3)	52.3 (32.0–65.8)	0.208
CE1 (%)^a^	82.7 (55.4–130.2)	70.1 (46.6–81.0)	0.097
Cell composition (×10^3^/μL)^b^			
CD3+	78.5 (57.5–131.1)	49.4 (16.1–74.8)	0.006
CD4+	55.1 (2.4–73.5)	23.9 (13.0–51.8)	0.038
CD8+	19.6 (7.5–60.3)	20.1 (2.2–31.1)	0.448
CD4/8 ratio	2.3 (0.7–9.1)	1.4 (0.6–5.8)	0.289
CD19+	6.5 (1.1–19.6)	3.0 (0.7–9.9)	0.187
NK cells	7.2 (2.8–13.4)	5.0 (2.2–16.1)	0.384
CD34+ subpopulations (per μL; %)			
MPP per μL	1211 (985–1674)	367 (174–636)	< 0.001
MPP %	52 (29–69)	52 (44–69)	0.291
LMPP per μL	530 (97–670)	136 (65–223)	< 0.01
LMPP %	20 (4–24)	19 (7–37)	0.201
EMP per μL	436 (236–1275)	200 (59–288)	0.013
EMP %	20 (11–49)	25 (15–37)	0.310
MLP + BLP per μL	152 (24–912)	16 (5–37)	0.035
MLP + BLP %	4 (1–27)	2 (1–4)	0.027
Late GMP per μL	74 (20–174)	11 (0–45)	< 0.01
Late GMP %	3 (1–9)	2 (0–4)	0.056
Colony forming units ×10^4^			
CFU‐GM^c^	63 (22–79)	28 (15–87)	< 0.05
BFU‐E^c^	48 (12–118)	23 (4–234)	0.143
CFU‐GEMM^d^	15 (0–42)	10 (2–56)	0.931
CD34+ ×10^6^/kg b.w. infused in 1st ASCT	4.0 (2.4–7.8)	2.7 (2.0–3.2)	< 0.001
Engraftment (day)			
ANC > 0.5 × 10^9^/L	10 (9–11)	11 (8–12)	0.405
Leukocytes > 1.0 × 10^9^/L	10 (9–11)	11 (9–14)	0.817
Platelets > 20 × 10^9^/L	13 (9–17)	12 (8–16)	0.9
Platelets > 50 × 10^9^/L	14 (11–28)	16 (14–29)	0.318

Abbreviations: ANC = absolute neutrophil counts, ASCT = autologous stem cell transplantation, *n* = number.

^a^ CE1 available in 18 pts. (9 plerixafor, 9 placebo).

^b^ Cell composition available in 17 pts. (8 plerixafor, 9 placebo).

^c^ CFU‐GM and BFU‐E available in 20 pts. (10 plerixafor, 10 placebo).

^d^ CFU‐GEMM available in 18 pts. (9 plerixafor, 9 placebo).

**FIGURE 1:**
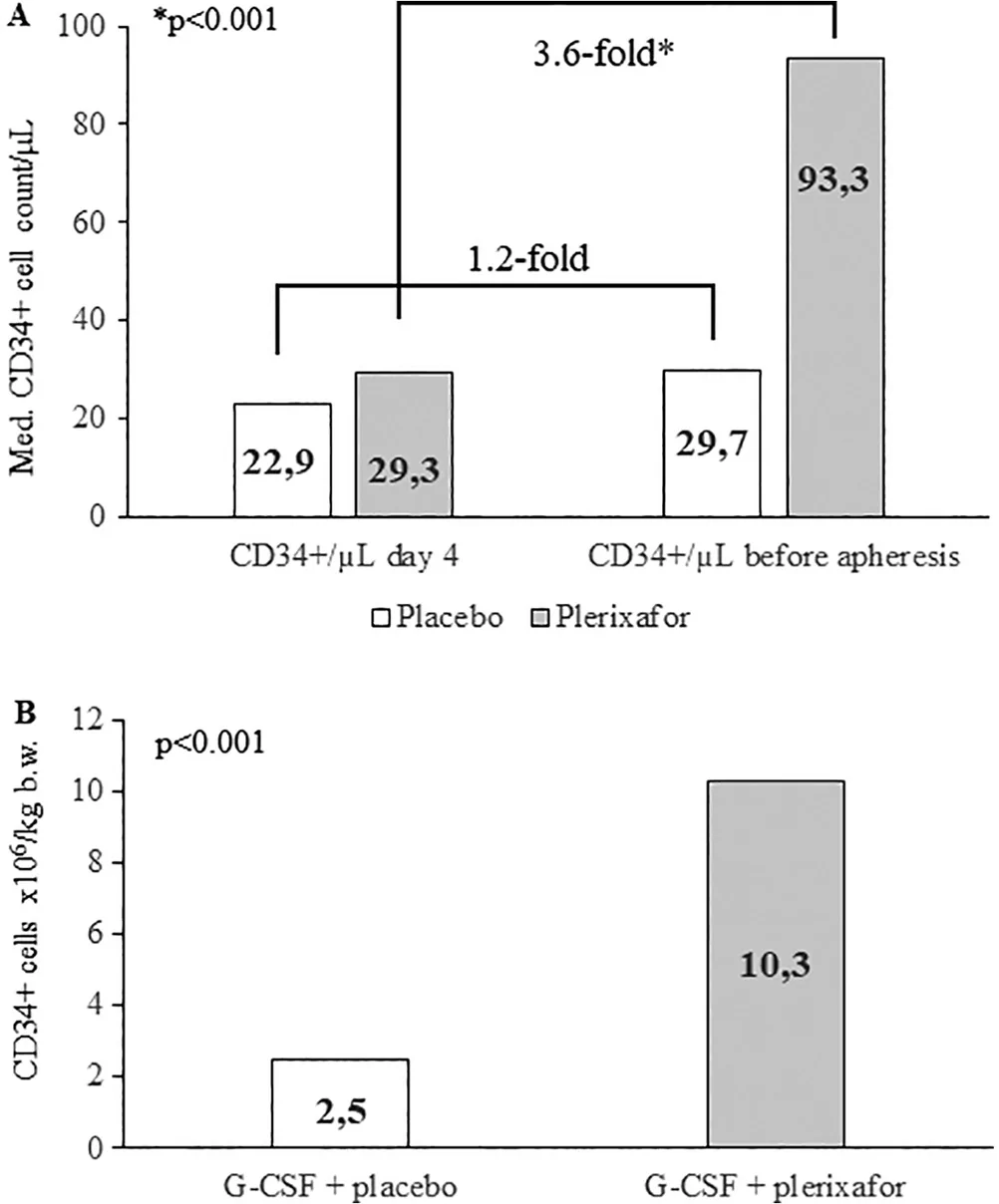
Peripheral blood CD34+ cell counts on day 4 and before apheresis after receiving either placebo or plerixafor (A) and CD34+ collection results in patients mobilized with G‐CSF and placebo or G‐CSF and plerixafor (B). Patients receiving placebo are depicted in white whereas patients receiving plerixafor are depicted in gray.

All patients in the plerixafor group reached the primary endpoint of ≥ 6 × 10^6^/kg CD34+ cells in one apheresis day versus none of patients in the placebo group, respectively (*p* < 0.001, Table 2, Figure 1B). The number of apheresis days required to collect ≥ 6 × 10^6^/kg CD34+ cells in the placebo group was a median of 2 (range, 2–3).

Patients treated with plerixafor collected a mean total of 10.4 × 10^6^/kg CD34 cells/kg (±1.9 SD; median, 10.3; range, 7.8–13.2), whereas placebo‐treated patients collected a mean total of 2.9 × 10^6^ CD34 cells/kg (±1.3 SD; median, 2.5; range, 2.9–1.3) (*p* < 0.001; Table 2, Figure 1). ce 1 and CE2 were similar between groups (Table 2). Owing to the small number of patients undergoing collection on day 4, a statistical comparison of CD34+ increase and collection outcomes between days 4 and 5 was not feasible (Table S1).

Cell composition of leukapheresis products was available from 8 plerixafor‐treated patients and 9 patients of the placebo cohort. Cell characterization of apheresis products showed significant higher CD3+ and CD4+ cells in plerixafor obtained HPC grafts, whereas no statistically significant difference was observed in the number of CD8+, CD19+, and NK‐cells and CD4/8 ratio (Table 2). In addition, plerixafor obtained HPC products contained significantly higher numbers of MPPs, LMPP, EMPs., and late GMPs. Plerixafor also mobilized significantly more MLP + BLP as measured in relative and absolute numbers. CFUs assays revealed significantly higher CFU‐GM in the plerixafor group (Table 2).

### 3.2 Transplantation and Engraftment

All patients underwent at least one transplantation and only 3 patients had a tandem transplantation (data not shown). The median number of CD34+ cells infused for the first ASCT was significantly higher in the plerixafor group (4.0 CD34+ cells × 10^6^/kg cells; range, 2.4–7.8 CD34+ cells × 10^6^/kg) as compared to the placebo group (2.7 CD34+ cells × 10^6^/kg; range, 2.0–3.2 CD34+ × 10^6^/kg). The engraftment was similar between groups and there were no graft failures observed. The median time to neutrophil and platelet engraftment was 10 and 13 days in the plerixafor group vs. 11 and 12 days in the placebo group, retrospectively (Table 2).

### 3.3 Graft Sustainability and Immunologic Engraftment

There were no significant differences observed in graft sustainability and immunologic recovery between the two groups analyzed on day 15, 28, and after 3 and 6 months (Table S2).

### 3.4 Safety

No severe adverse events were observed. The overall incidence of adverse events was similar between the 2 groups, 4/10 patients (40%) in the plerixafor and 5/11 patients (45.5%) in the placebo group experienced adverse events. Adverse events associated with the mobilization regimen were recorded in 9/21 patients (43%) and consisted of mild to moderate diarrhea (*n* = 2), mild to moderate cephalea (*n* = 3), moderate joint and muscle pain (*n* = 2), mild to moderate bone pain (*n* = 6), and fatigue (*n* = 2). Five patients reported more than one adverse event (Table S3).

## 4 Discussion

This randomized, double‐blind, placebo‐controlled study confirms that the addition of plerixafor to G‐CSF significantly improves stem cell mobilization in patients with MM who exhibit moderate mobilization potential. All patients in the plerixafor group achieved the primary endpoint of ≥ 6.0 × 10^6^ CD34^+^ cells/kg in a single apheresis, while none in the placebo group met this threshold, underlining the superior efficacy of plerixafor even in patients who were not poor mobilizers by classical definitions [23]. These findings are consistent with earlier reports, such as DiPersio et al., who demonstrated improved mobilization success rates using plerixafor in combination with G‐CSF compared to G‐CSF alone [12].

Notably, the observed median CD34^+^ cell yield in the plerixafor group (10.3 × 10^6^ CD34+ cells/kg) exceeded the target for tandem transplantation, while patients in the placebo arm had substantially lower yields (2.52 × 10^6^ CD34+ cells/kg), requiring multiple apheresis sessions. In particular, in 5 (45%) patients in the placebo group less than 2.5 × 10^6^ CD34+ cells/kg b.w. could be collected. This difference in yield occurred despite similar baseline PB CD34^+^ levels between groups on day 4 of G‐CSF, highlighting the specific effect and benefit of plerixafor on stem cell mobilization also in moderate mobilizers. As observed in the pivotal study, we can confirm that the increase of CD34+ cells/μL from day 4 to day 5 in the placebo group was less than 1.5 fold.

The original trial design anticipated enrollment of 90 patients; however, accrual was terminated earlier after inclusion of only 21 patients. Besides the COVID‐19 pandemic, the main limiting factor was the widespread adoption of daratumumab‐containing induction regimens for newly diagnosed MM patients during the study period. Daratumumab, a CD38‐targeting moAb, has been increasingly used as part of standard induction therapy, particularly following the CASSIOPEIA trial results [8]. Several studies, including Eleutherakis Papaiakovou et al. have reported that daratumumab impairs stem cell mobilization, reduces CD34^+^ yield, and increases the need for plerixafor [10, 11]. This likely contributed to a higher proportion of patients failing to meet our strict inclusion criterion of 15–40 CD34^+^ cells/μL on day 4 of G‐CSF.

Beyond total CD34^+^ counts, our study revealed significant differences in graft composition after use of plerixafor. Flow cytometric analyses demonstrated that plerixafor mobilized not only higher numbers of CD34^+^ cells but also enriched for early progenitor subsets, including MPPs, LMPPs, and EMPs. These subsets represent more primitive hematopoietic populations and have been associated with superior long‐term hematopoietic reconstitution potential [17]. In contrast, the placebo group had markedly lower absolute numbers of these subpopulations, especially MPPs and MLP + BLPs. Interestingly, previous studies have demonstrated that poor mobilizers not only exhibit lower overall CD34^+^ cell yields, but also show distinct shifts in progenitor subtypes. Specifically, MPPs and EMPs—which are early and broadly potent—are significantly reduced in poor mobilizers, whereas more lineage‐restricted populations such as LMPPs and late GMPs appear elevated, possibly reflecting reduced content of hematopoietic progenitor cells in the bone marrow (i.e., bone marrow insufficiency) [17]. In contrast, good mobilizers typically display a predominance of primitive subtypes, indicative of a more balanced and robust mobilization response. We can show that MM patients considered to be moderate mobilizers based on their PB CD34+ counts on day 4 of G‐CSF were able to mobilize more primitive HPCs such as MPPs, EMPs, and LMPPs, after administration of plerixafor. These findings suggest that in moderate mobilizers, plerixafor is able to not only increase the overall CD34^+^ cell yield but also to broaden mobilization from bone marrow into the PB across hematopoietic hierarchies. This enhanced diversity of progenitor cells may be particularly relevant for achieving durable engraftment in transplant settings, where functional reserve is critical.

Colony‐forming unit assays further supported the observed phenotypic differences between the mobilized grafts. CFU‐GM numbers were significantly higher in the plerixafor group, suggesting enhanced functional potency of the collected progenitor cell products. Although BFU‐E and CFU‐GEMM counts did not differ significantly, the overall clonogenic potential of the grafts, as reflected by CFU‐GM formation (Table 2), was greater in patients receiving plerixafor. These findings are consistent with previous reports demonstrating that plerixafor preferentially mobilizes functionally robust and primitive hematopoietic progenitor cells.

Beyond ensuring timely hematopoietic recovery after ASCT, the collection of grafts with a higher functional progenitor cell content may also be clinically relevant in the current treatment landscape of multiple myeloma. Sustained hematopoietic reserve following transplantation may facilitate the delivery of post‐transplant consolidation and maintenance therapies, which have become integral components of modern myeloma treatment strategies. While the present study was not designed to assess long‐term hematopoietic fitness, the higher clonogenic activity observed in the plerixafor‐mobilized grafts raises the possibility that qualitative differences in graft composition may contribute to improved hematopoietic resilience after transplantation.

Importantly, despite these quantitative and qualitative differences in the grafts, clinical outcomes post‐transplant including neutrophil and platelet engraftment, graft sustainability, and immune reconstitution were comparable between groups. These findings indicate that while plerixafor substantially improves mobilization efficiency and increases graft cellularity, G‐CSF alone can still yield clinically effective grafts in a proportion of moderately mobilizing patients, albeit often at the expense of additional apheresis procedures.

In the current treatment landscape, the collection of sufficient CD34+ cells beyond the minimum dose required for a single ASCT may remain clinically relevant. With the increasing use of anti‐BCMA‐directed CAR‐T cell therapies such as idecabtagene vicleucel and ciltacabtagene autoleucel in relapsed/refractory MM, prolonged and/or recurrent cytopenias have emerged as important toxicities. Both product labels describe prolonged cytopenias and the potential need for stem cell support for hematopoietic recovery. Moreover, clinical reports indicate that autologous CD34+ stem cell boosts can be an effective rescue strategy for delayed hematopoietic recovery after anti‐BCMA‐directed CAR‐T cell therapy. Therefore, achieving higher CD34+ cell yields during initial mobilization may provide an important hematopoietic reserve, not only for tandem ASCT but also for future cellular therapies and their potential complications [24, 25, 26, 27].

From an economic perspective, the higher acquisition cost of plerixafor must be balanced against the costs and risks associated with repeated apheresis procedures. Additional collections require further use of apheresis facilities, personnel, and disposable kits and may prolong or necessitate additional hospital visits. They also increase patient burden and cumulative exposure to procedure‐related adverse events, including citrate‐related symptoms, venous‐access complications, hemodynamic changes, and thrombocytopenia. Conversely, plerixafor administration entails additional drug costs and is associated predominantly with mild adverse effects, such as gastrointestinal symptoms and injection‐site reactions. Thus, in intermediate mobilisers, the ability of plerixafor to achieve the collection target with fewer procedures may partly offset its acquisition cost while reducing patient burden. Nevertheless, a formal health‐economic analysis was not performed, and cost‐effectiveness will depend on local drug prices, reimbursement arrangements, and apheresis‐related costs.

While our approach requires additional coordination and real‐time assessment of peripheral blood CD34^+^ cell counts, it allows identification of a patient population that is frequently overlooked by current mobilization algorithms. The finding that all plerixafor‐treated patients achieved collection targets sufficient for tandem transplantation in a single short apheresis session suggests that the benefit of plerixafor may extend beyond the traditional poor mobilizer population. Moreover, the evolving treatment landscape, particularly the increased use of daratumumab, restricted recruitment and reflects the challenge of conducting randomized trials in rapidly changing therapeutic environments.

Our study has several limitations. First, the small sample size limits its statistical power and the generalizability of the findings. Second, variation in the timing of apheresis—performed on either day 4 or day 5 of G‐CSF mobilization—precluded a meaningful comparison of mobilization results and collection outcomes between these time points.

## 5 Conclusion

In conclusion, plerixafor significantly improved stem cell mobilization efficiency in MM patients with intermediate mobilization capacity, enabling successful collection of sufficient CD34^+^ cells for tandem ASCT within a single apheresis session while enriching grafts with primitive and functionally competent hematopoietic progenitors. Although post‐transplant engraftment outcomes were comparable, the superior graft quality achieved with plerixafor may provide clinically relevant hematopoietic reserve for future transplant strategies and cellular therapies. Larger prospective studies are warranted to define individualized, risk‐adapted mobilization algorithms incorporating plerixafor beyond the traditional poor mobilizer population.

## Author Contributions

W.N. planned the study, performed data analysis and drafted the manuscript; T.A. planned the study, performed data analysis, Ku.M. performed data analysis and drafted the manuscript; H.‐K.B. and N.P. enrolled patients and provided clinical data, R.K. and S.N. contributed to methods, provided data and analyzed stem cell cultures; B.M., K.V., K.E., R.R., A.H., Kr.M., R.W. enrolled patients and provided clinical data, K.B. and O.S. analyzed data, S.M. enrolled patients, provided clinical data and wrote the manuscript, G.H.T. planned the study, enrolled patients, provided clinical data and wrote the manuscript. All authors critically reviewed the manuscript, approved the final version, and agreed to be accountable for all aspects of the work.

## Funding

This investigator‐initiated study received financial support from Sanofi Genzyme. Sanofi Genzyme had no role in data analysis, interpretation, or manuscript preparation.

## Ethics Statement

This prospective, randomized, placebo‐controlled, double‐blinded study was conducted in accordance with the ethical standards of the institutional research committee and with the 1964 Helsinki Declaration and its later amendments. The study protocol met institutional review board (IRB) exemption criteria at Medical University of Vienna (EK 2274/2016).

## Consent

All patients gave informed consent to participate in this study.

## Conflicts of Interest

W.N. receives research support from Sanofi Genzyme and speaker's fees from BMS Celgene, Johnson & Johnson, Kite Gilead, Miltenyi Biotec, Sanofi Genzyme, Therakos, Vertex; S.M. receives consultancy/speaker's fees from Johnson & Johnson, Sanofi, Pfizer, AbbVie and Bristol‐Myers Squibb; H.‐K.B. receives speaker‘s fees from BMS Celgene, Johnson & Johnson, Kite Gilead; N.P. received speaker's fees from Amgen, Celgene BMS, Janssen, GSK, Sanofi, Roche, Astra Zeneca, Pfizer, and Takeda; R.W. reports honoraria from BMS Celgene and Kite Gilead; A.H. receives speaker's fees from Amgen, Astra Zeneca, BMS Celgene, GlaxoSmithKline, Johnson & Johnson (including research support), Pfizer, Sanofi Genzyme, Takeda; Kr.M. reports Ad boards/consultancy fees from Amgen, BMS Celgene, J&J, Sanofi, Takeda, GlaxoSmithKline, Pfizer, Oncopeptides, Stemline, Abbvie, Gilead Kite; T.G.H. reports honoraria from Therakos, Takeda, Sanofi and Stemline; T.A., Ku.M., R.K., S.N., B.M., K.V., O.S., K.B., K.E., and R.R. declare no conflicts of interest.

## Supporting information


**Table S1:** CD34+ characteristics of 1st collections on Day 4 or Day 5 of G‐CSF.
**Table S2:** Graft sustainability and immunologic recovery.
**Table S3:** Summary of patients with adverse events.
**Figure S1:** Schematic Study overview.

## Data Availability

The data that support the findings of this study are available on request from the corresponding author. The data are not publicly available due to privacy or ethical restrictions.

## References

[1] S. V. Rajkumar , “Multiple Myeloma: 2024 Update on Diagnosis, Risk‐Stratification, and Management,” American Journal of Hematology 99 (2024): 1802–1824. 10.1002/ajh.27422 PMC11404783 38943315

[2] A. Palumbo and K. Anderson , “Multiple Myeloma,” New England Journal of Medicine 364 (2011): 1046–1060. 10.1056/NEJMra1011442 21410373

[3] N. Grieb , A. Oeser , M. Ferle , et al., “Single Versus Tandem Autologous Stem Cell Transplantation in Newly Diagnosed Multiple Myeloma,” Bone Marrow Transplantation 60 (2025): 335–345. 10.1038/s41409-024-02490-1 PMC11893441 39638882

[4] D. Galligan , S. Williamson , J. Myers , et al., “Second Autologous Stem Cell Transplant as Salvage in Multiple Myeloma ‐ The Oregon Health and Science University Experience,” Clinical Lymphoma, Myeloma & Leukemia 22 (2022): 105–112. 10.1016/j.clml.2021.08.008 34686447

[5] A. Perrot , J. Lambert , C. Hulin , et al., “Measurable Residual Disease–Guided Therapy in Newly Diagnosed Myeloma,” New England Journal of Medicine 393 (2025): 425–437. 10.1056/NEJMoa2505133 PMC13292240 40459097

[6] F. Malard , N. Kröger , I. H. Gabriel , et al., “Plerixafor for Autologous Peripheral Blood Stem Cell Mobilization in Patients Previously Treated With Fludarabine or Lenalidomide,” Biology of Blood and Marrow Transplantation 18 (2012): 314–317. 10.1016/j.bbmt.2011.10.003 22001752

[7] M. Mohty , K. Hübel , N. Kröger , et al., “Autologous Haematopoietic Stem Cell Mobilisation in Multiple Myeloma and Lymphoma Patients: A Position Statement From the European Group for Blood and Marrow Transplantation,” Bone Marrow Transplantation 49 (2014): 865–872. 10.1038/bmt.2014.39 24686988

[8] P. Moreau , M. Attal , C. Hulin , et al., “Bortezomib, Thalidomide, and Dexamethasone With or Without Daratumumab Before and After Autologous Stem‐Cell Transplantation for Newly Diagnosed Multiple Myeloma (CASSIOPEIA): A Randomised, Open‐Label, Phase 3 Study,” Lancet 394 (2019): 29–38. 10.1016/S0140-6736(19)31240-1 31171419

[9] P. Sonneveld , M. A. Dimopoulos , M. Boccadoro , et al., “Daratumumab, Bortezomib, Lenalidomide, and Dexamethasone for Multiple Myeloma,” New England Journal of Medicine 390 (2024): 301–313. 10.1056/NEJMoa2312054 38084760

[10] C. Röllig , S. Knop , and M. Bornhäuser , “Multiple myeloma,” Lancet 385 (2015): 2197–2208. 10.1016/S0140-6736(14)60493-1 25540889

[11] E. Eleutherakis Papaiakovou , E. Terpos , N. Kanellias , et al., “Impact of Daratumumab on Stem Cell Mobilization and Collection, Engraftment and Early Post‐Transplant Complications Among Multiple Myeloma Patients Undergoing Autologous Stem Cell Transplantation,” Leukemia & Lymphoma 64 (2023): 2140–2147. 10.1080/10428194.2023.2253479 37655597

[12] J. F. DiPersio , E. A. Stadtmauer , A. Nademanee , et al., “Plerixafor and G‐CSF Versus Placebo and G‐CSF to Mobilize Hematopoietic Stem Cells for Autologous Stem Cell Transplantation in Patients With Multiple Myeloma,” Blood 113 (2009): 5720–5726. 10.1182/blood-2008-08-174946 19363221

[13] S. Giralt , L. Costa , J. Schriber , et al., “Optimizing Autologous Stem Cell Mobilization Strategies to Improve Patient Outcomes: Consensus Guidelines and Recommendations,” Biology of Blood and Marrow Transplantation 20 (2014): 295–308. 10.1016/j.bbmt.2013.10.013 24141007

[14] European Medicines Agency (EMA) , “Plerixafor (Mozobil): Summary of Product Characteristics,” (2016), https://ec.europa.eu/health/documents/community‐register/2016/20160302134414/anx_134414_en.pdf.

[15] P. Vishnu , V. Roy , A. Paulsen , and A. C. Zubair , “Efficacy and Cost‐Benefit Analysis of Risk‐Adaptive Use of Plerixafor for Autologous Hematopoietic Progenitor Cell Mobilization,” Transfusion 52 (2012): 55–62. 10.1111/j.1537-2995.2011.03206.x 21658047

[16] J. Li , E. Hamilton , L. Vaughn , et al., “Effectiveness and Cost Analysis of ‘Just‐In‐Time’ Salvage Plerixafor Administration in Autologous Transplant Patients With Poor Stem Cell Mobilization Kinetics,” Transfusion 51 (2011): 2175–2182. 10.1111/j.1537-2995.2011.03136.x 21492180

[17] N. Worel , N. Frank , C. Frech , and G. Fritsch , “Influence of Plerixafor on the Mobilization of CD34+ Cell Subpopulations and Lymphocyte Subtypes,” Transfusion 57 (2017): 2206–2215. 10.1111/trf.14182 28580637

[18] N. Worel , G. Fritsch , H. Agis , et al., “Plerixafor as Preemptive Strategy Results in High Success Rates in Autologous Stem Cell Mobilization Failure,” Journal of Clinical Apheresis 32 (2017): 224–234. 10.1002/jca.21496 27578390

[19] J. Dmytrus , S. Matthes‐Martin , H. Pichler , et al., “Multi‐Color Immune‐Phenotyping of CD34 Subsets Reveals Unexpected Differences Between Various Stem Cell Sources,” Bone Marrow Transplantation 51 (2016): 1093–1100. 10.1038/bmt.2016.88 27042837

[20] B. Wognum , N. Yuan , B. Lai , and C. L. Miller , Colony Forming Cell Assays for Human Hematopoietic Progenitor Cells, vol. 946 (Humana press, 2013), 267–283, 10.1007/978-1-62703-128-8_17. 23179838

[21] G. Calandra , J. McCarty , J. McGuirk , et al., “AMD3100 Plus G‐CSF Can Successfully Mobilize CD34+ Cells From Non‐Hodgkin's Lymphoma, Hodgkin's Disease and Multiple Myeloma Patients Previously Failing Mobilization With Chemotherapy and/or Cytokine Treatment: Compassionate Use Data,” Bone Marrow Transplantation 41 (2008): 331–338. 10.1038/sj.bmt.1705908 17994119

[22] K. Hübel , M. M. Fresen , H. Salwender , et al., “Plerixafor With and Without Chemotherapy in Poor Mobilizers: Results From the German Compassionate Use Program,” Bone Marrow Transplantation 46 (2011): 1045–1052. 10.1038/bmt.2010.249 20972470

[23] A. Olivieri , M. Marchetti , R. Lemoli , et al., “Proposed Definition of ‘Poor Mobilizer’ in Lymphoma and Multiple Myeloma: An Analytic Hierarchy Process by Ad Hoc Working Group Gruppo ItalianoTrapianto di Midollo Osseo,” Bone Marrow Transplantation 47 (2012): 342–351. 10.1038/bmt.2011.82 PMC3296914 21625224

[24] M. Mohan , A. Szabo , A. Patwari , et al., “Autologous Stem Cell Boost Improves Persistent Immune Effector Cell Associated Hematotoxicity Following BCMA Directed Chimeric Antigen Receptor T (CAR T) Cell Therapy in Multiple Myeloma,” Bone Marrow Transplantation 59 (2024): 647–652. 10.1038/s41409-024-02233-2 38361116

[25] T. Jain , T. S. Olson , and F. L. Locke , “How I Treat Cytopenias After CAR T‐Cell Therapy,” Blood 141 (2023): 2460–2469. 10.1182/blood.2022017415 PMC10646792 36800563

[26] C. Varga , M. Robinson , J. A. Davis , et al., “Efficacy of Stem Cell Boost (SCB) for Chimeric Antigen Receptor‐T Cell Therapy (CAR‐T)‐Related Hematologic Toxicity in Patients With Relapsed/Refractory Multiple Myeloma (RRMM)‐Real World Experience From the US Multiple Myeloma Immunotherapy Consortium,” Blood Cancer Journal 16 (2026): 39, 10.1038/s41408-026-01469-z. PMC13018517 41862439

[27] K. Rejeski , M. Subklewe , M. Aljurf , et al., “Immune Effector Cell–Associated Hematotoxicity: EHA/EBMT Consensus Grading and Best Practice Recommendations,” Blood 142 (2023): 865–877. 10.1182/blood.2023020578 37300386

