# Supplementary material for: Plerixafor and G‐CSF Versus Placebo and G‐CSF in Moderately Mobilizing Patients With Multiple Myeloma to Optimize Hematopoietic Stem Cell Collection Results (OPTIMIZE)
Source: J Clin Apher. 2026 Oct 12;41(5):e70188. doi: 10.1002/jca.70188 (PMC13658576; doi:10.1002/jca.70188)
Supplement: Supplementary file 1 [file JCA-41-e70188-s001.docx]

Supplementary Material

**Plerixafor and G-CSF versus placebo and G-CSF in moderately mobilizing patients with multiple myeloma to optimize hematopoietic stem cell collection results (OPTIMIZE)**

The supplementary material contains detailed information on how the flow cytometry analyses and the stem cell assay analyses were performed and calculated. Supplementary table 1 describes in detail the CD34+ characteristics of 1^st^ collections performed on on Day 4 or Day 5 of G-CSF and supplementary table 2 shows graft sustainability and immunologic recovery up to 6 months after autologous stem cell transplantation. Supplementary figure 1 depicts the flow chart of the clinical study.

***Flow cytometry***

Blood cell counts were obtained from a hematology analyzer (KX-21N, Sysmex Corporation). Samples were diluted with Dulbecco's phosphate-buffered saline (Life Technologies) to adjust white blood cell (WBC) numbers to fewer than 40 x 10^9^ cells/L before immune staining. All monoclonal antibodies (MoAbs) were used at pretested concentrations, and compensations had been set accordingly. Isotype controls and fluorescence-minus-one analyses were used to define gating and compensation. Analyses were performed on a flow cytometer (FACS Fortessa, BD Biosciences) equipped with four solid-state lasers with excitation wave lengths (nm) of 488, 405, 561, and 640. Quality controls were performed at least once weekly with research beads (CS&T, BD Biosciences). Computer software (FACSDiVa 6, BD Biosciences) was used for data acquisition and evaluation. For each analysis 150,000 CD45+ events were acquired.

*Single platform protocol*

The MoAb cocktail for defining the CD34+ subpopulations contained CD7 PeCF594, CD10 BV421, CD33 APC-R700, CD38 PE-Cy7, CD45RA BV510 (all from BD Biosciences), CD3 PerCPeFl710 (eBiosciences), CD19 APC-Cy7 (BD PharMingen), and AC133-1 APC (Miltenyi Biotec; see Table S1 [available as supporting information in the online version of this paper] for detailed antibody cocktail composition). For CD34+ subgroup analyses, 100 µL of the cell sample was reverse-pipetted into a tube (Trucount, BD Biosciences). After the stem cell enumeration kit (20 µL of 7-AAD and 20 µL of CD45 FITC/CD34 PE; BD Biosciences) and the MoAb cocktail (25.5 µL) were added, cells were mixed and incubated light-shielded at room temperature (20-25°C) for 20 minutes. Red blood cells (RBCs) were lysed by adding 2 mL of ammonium chloride solution (BD Biosciences) for 10 minutes before samples were analyzed.

The MoAb cocktail for defining lymphocyte subsets comprised CD19 BV510, TCR-γδ PE, CD45 PerCP, CD56 PE-Cy7, and CD8 APC-Cy7 (all from BD Biosciences), TCR-αβ FITC and CD3 PE-Texas Red (both from BD PharMingen), and CD4 PerCP e-FL710 and CD16 APC (both from eBiosciences; Table S1). For analyses of lymphocytes, 10 µL of the cell sample was reverse-pipetted into a tube (Trucount, BD Biosciences). After 8.3 µL of the MoAb cocktail was added, cells were mixed, incubated light-shielded at room temperature for 20 minutes, and then diluted 1:10 with ammonium chloride solution (BD Biosciences). After RBC lysis, samples were analyzed.

*Gating strategy*

To define CD34+ cells and their subsets, we used the gating strategy described previously.^16^ Briefly, viable hematopoietic progenitor cells (HPCs) were determined by their positivity for CD34, their weak expression of CD45, their typical position in the lymphomonocytic area of the forward scatter/side scatter (FSC/SSC) dot plot and their negativity for 7-AAD.^16^ CD34 subtyping was started with CD45RA, as this marker allows the definition of two distinct subgroups separating earlier CD45RA− from more committed CD45RA+ cells. These subpopulations were then further evaluated in CD133 versus CD10 contour plots where they formed at least six distinct CD34 subpopulations (see Fig. S1 [available as supporting information in the online version of this paper] for gating strategy of CD34+ subsets). The resulting subpopulations were examined for their expression of CD38. Beads were double-gated in two different dot plots (APC vs. SSC and APC-Cy7 vs. FITC) to exclude false-positive events.

The gating strategy for lymphocyte subsets was identical to the method described elsewhere. ^18^ Briefly, viable WBCs were first defined by their CD45 expression and their typical position in the FSC/SCC dot plot. In a next step, CD3+ T cells, CD19+ B cells, and CD56+ NK cells were defined. CD3+CD56– T cells were subdivided into γ-δ and α-β T cells, the latter forming the CD4+ and CD8+ T-cell subtypes. CD56+ NK-cells were negative for CD3 and CD19 and expressed CD16.

The following formula was used to calculate the number of target cells/µL:

Absolute cell numbers per kg body weight (×10^6^) were then calculated:

***Stem cell culture assay***

The in vitro stem cell assays were performed on a thawed reference vial within 72 h of cryopreservation as quality control for the freezing thawing process. A commercially available kit (MethoCult^TM^ GF H84434, Stemcell Technologies, Vancouver, Canada) was used according to the manufacturer´s instruction.^20^

The kit consists of Iscove's MDM, methylcellulose, fetal bovine serum, bovine serum albumin, recombinant human (rh) stem cell factor, recombinant human granulocyte-macrophage colony-stimulating factor (rhGM-CSF), recombinant human granulocyte colony-stimulating factor (rhG-CSF), rh-interleukin 3, and rh erythropoietin (EPO). The cryopreserved stem cell product reference vial was thawed and diluted 1:100 with buffered salt solution/acid-citrate-dextrose. The calculated volume of the dilution with 2x10^4^ viable nucleated cells (NC) was plated together with the reagents under aseptic conditions. The plates were incubated for 14 days in an incubator (37°C, 5% CO2, ≥ 95% humidity).

An inverted microscope was used for evaluation of the colony forming unit-granulocyte-macrophage (CFU-GM), burst forming unit-erythroid (BFU-E) and colony forming unit-granulocyte erythrocyte macrophage megakaryocyte (CFU-GEMM) colonies with a 40-fold magnification. The mean count of a double was used.

The following formula for calculation was used:

| CFU-GM (10^4^/kg) = | CFU-GM count X plated NC (10^9^/l) x Vol of the cryo-product (ml) x 100 |
| --- | --- |
|  | 20.000 x patient´s weight (kg) |

The results of CFU-GM and CFU-GEMM are summed up.

The proliferative capacity (PC) was calculated as follows:

| PC (%) = | (CFU-GM + CFU-GEMM) x 10^4^/kg |
| --- | --- |
|  | CD34 x 10^6^/kg |

A proliferative capacity of 5-10% was considered to be sufficient. The lab participated in a proficiency testing program of Stem Cell Technologies on a regular basis.

**Supplementary Table 1.** CD34+ characteristics of 1^st^ collections on Day 4 or Day 5 of G-CSF

| Data given in median, range /where applicable) | **Plerixafor** | | **Placebo** | |
| --- | --- | --- | --- | --- |
| Collection on day of G-CSF | **Day 4** (n=3) | **Day 5** (n=7) | **Day 4** (n=2) | **Day 5** (n=9) |
| Peripheral blood CD34+ cells/µL  Before plerixafor/placebo  Before first apheresis | 34.8 (32.5-34.8)  95.4 (85.3-95.5) | 25.0 (16.9-34.0)  91.2 (53.4-140) | 21.36; 29.16  23.4; 33.8 | 22.0 (15.3-38.4)  29.3 (11.0-56.2) |
| X-fold increase of CD34+ cells/µL in peripheral blood | 2.7 (2.6-2.9) | 3.4 (2.2-6.4) | 1.1; 1.2 | 1.3 (0.5-2.9) |
| CD34+ cells x 10^6^/kg in the 1^st^ leukapheresis | 10.4 (7.8-10.9) | 10.2 (8.1-13.2) | 2.4; 3.1 | 2.52 (1.4-5.3) |
| Collection efficiency 2 (%) | 54.9 (42.4-65.4) | 59.2 (40.3-93.3) | 52.2; 57.2 | 52.3 (32.0-65.8) |

**Supplementary Table 2.** Graft sustainability and immunologic recovery

| Data given in median, range | **Day 15 after ASCT** | **Day 28 after ASCT** | **3 months after ASCT** | **6 months after ASCT** |
| --- | --- | --- | --- | --- |
| Plerixafor group  WBC x10^9^/L  Hemoglobin g/dL  Platelets x10^9^/L  CD3+ x 10^3^/uL  CD4+ x 10^3^/uL  CD8+ x 10^3^/uL  CD19+ x 10^3^/uL  NK-cells x 10^3^/uL | 9.25 (5.83-14.8)  11.5 (9.2-13.2)  13 (13-120)  868 (129-1470)  484 (82-1077)  355 (173-473)  11 (2-57)  332 (9-933) | 6.52 (3.7-10.7)  12.7 (11.1-14.8)  213 (109-324)  1342 (260-2079)  293 (177-558)  470 (68-1370)  16 (2-237)  315 (196-1513) | 5.55 (3.77-6.91)  13.6 (11.6-14.6)  213 (111-313)  956 (320-2130)  360 (93-504)  345 (110-1724)  157 (32-822)  222 (6-899) | 5.65 (3.0-7.0)  13.45 (11.0-16.0)  232 (104-323)  1577 (1197-2214)  341 (298-615)  623 (248-1554)  84 (38-456)  251 (43-318) |
| Placebo group  WBC x10^9^/L  Hemoglobin g/dL  Platelets x10^9^/L  CD3+ x 10^3^/µL  CD4+ x 10^3^/µL  CD8+ x 10^3^/µL  CD19+ x 10^3^/µL  NK-cells x 10^3^/µL | 7.28 (1.84-11.54)  9.8 (8.1-14.4)  46 (24-130)  583 (370-819)  279 (193-422)  98 (53-397)  10 (3-3614)  334 (142-528) | 5.56 (4.1-9.29)  11.8 (10.5-16.9)  229 (101-423)  970 (455-2602)  265 (228-540)  678 (205-1768)  13 (6-42)  424 (183-863) | 5.60 (3.47-7.47)  12.4 (11.2-16.1)  184 (128-327)  778 (308-2077)  291 (107-536)  393 (50-1454)  210 (0-400)  147 (52-1053) | 4.40 (2.0-6.0)  13.1 (10-16.0)  161 (124-282)  742 (361-1109)  323 (202-471)  343 (120-519)  251 (22-492)  103 (92-531) |

ASCT=autologous stem cell transplantation, WBC=white blood cells

**Supplementary Table 3:** Summary of patients with adverse events

| Data given in n (%) | **Plerixafor group (n=10)** | **Placebo group (n=11)** | **Total (n=21)** |
| --- | --- | --- | --- |
| Any AE | 4 (40%) | 5 (45.5%) | 9 (43%) |
| Diarrhea  Mild  Moderate | 0  1 (10%) | 1 (9%)  0 | 1 (4.8%)  1 (4.8%) |
| Cephalea  Mild  Moderate | 0  0 | 2 (18%)  1 (9%) | 2 (9.5%)  1 (4.8%) |
| Joint and muscle pain  Mild  Moderate | 0  1 | 0  1 | 0  2 (9.5%) |
| Bone pain  Mild  Moderate | 1 (10%)  2 (20%) | 1 (9%)  2 (18%) | 2 (9.5%)  4 (19%) |
| Fatigue  Mild  Moderate | 1 (10%)  0 | 0  1 (9%) | 1 (4.8%)  1 (4.8%) |

AE= adverse event, n= number of subjects with event, percentages are based on n.

**Supplementary figure 1:** Schematic Study overview

^1^Plerixafor or placebo dispensed by the hospital pharmacy
